# Radiation-Induced Thyroid Cancers: Overview of Molecular Signatures

**DOI:** 10.3390/cancers11091290

**Published:** 2019-09-02

**Authors:** Keiji Suzuki, Vladimir Saenko, Shunichi Yamashita, Norisato Mitsutake

**Affiliations:** 1 Department of Radiation Medical Sciences, Atomic Bomb Disease Institute, Nagasaki University, 1-12-4 Sakamoto, Nagasaki 852-8523, Japan; 2 Fukushima Medical University, 1 Hikariga-oka, Fukushima, Fukushima 960-1295, Japan; 3 Center for Advanced Radiation Emergency Medicine at the National Institutes for Quantum and Radiological Science and Technology, 4-9-1 Anagawa, Inage-ku, Chiba, Chiba 263-8555, Japan

**Keywords:** radiation, thyroid carcinoma, RET/PTC, driver mutation, molecular signature

## Abstract

Enormous amounts of childhood thyroid cancers, mostly childhood papillary thyroid carcinomas (PTCs), after the Chernobyl nuclear power plant accident have revealed a mutual relationship between the radiation exposure and thyroid cancer development. While the internal exposure to radioactive ^131^I is involved in the childhood thyroid cancers after the Chernobyl accident, people exposed to the external radiation, such as atomic-bomb (A-bomb) survivors, and the patients who received radiation therapy, have also been epidemiologically demonstrated to develop thyroid cancers. In order to elucidate the mechanisms of radiation-induced carcinogenesis, studies have aimed at defining the molecular changes associated with the thyroid cancer development. Here, we overview the literatures towards the identification of oncogenic alterations, particularly gene rearrangements, and discuss the existence of radiation signatures associated with radiation-induced thyroid cancers.

## 1. Introduction

Radiation exposure has been well documented to take part in cancer development in the human body. Increased risks in a variety of cancer mortality/incidences, including the thyroid cancer incidence, have been demonstrated among the Life Span Study (LSS) cohort of the A-bomb survivors in Hiroshima and Nagasaki [1,2]. The accident in the Chernobyl nuclear power plant (CNPP), which released large amounts of radioactive materials into the environment, has caused the excess cases of thyroid cancers among children living in the contaminated areas nearby the CNPP [3,4,5,6]. Epidemiological studies have indicated an apparent dose-dependent induction of thyroid cancers, confirming that the radiation exposure is the primary cause of thyroid cancer induction [7,8,9]. Other examples include the increased thyroid cancer incidence in the patients who received medical radiation therapy for diseases, such as tinea capitis, enlarged thymus glands, and tonsils [10]. Thus, radiation-induced thyroid cancers have provided unequalled examples to unveil the molecular mechanisms of radiation-induced carcinogenesis, as well as a role of radiation exposure in thyroid carcinogenesis.

## 2. Childhood Thyroid Cancers after the Chernobyl Accident

During the accident at the CNPP on 26 April 1986, a large amount of radioactive materials were released into the environment leading to the radiation exposure of some 5 million of residents in the most affected areas of Ukraine, Belarus, and Russia [3,4,5,6]. In particular, the fallout of radioactive iodine resulted in significant internal exposures in children mainly through the ingestion of contaminated milk. As a result, unprecedentedly high numbers of childhood thyroid cancer have been diagnosed, which are the main health effects of the accident in the population [11,12,13]. Four to five years after the accident, excess cases of childhood thyroid cancers were first reported. Thyroid cancer cases were particularly profound among the youngest children aged 0–5 years at exposure, while no such dramatic increase was observed in the adults. Between 1991 and 2005, 6848 cases were diagnosed among those exposed at the age under 18 years in 1986, and according to the recent WHO report update, more than 11,000 thyroid cancer cases were documented by 2016 in the individuals exposed during childhood in the three affected countries [14]. Note that the incidence of thyroid cancer in children born after the Chernobyl accident was significantly lower, almost the background level, indicating that the considerable increase in childhood thyroid cancer cases was evidently due to the internal exposure to the radioactive iodine [3,4,9,15].

The relationship between the internal exposure to radiation (β-rays and γ-rays) from ^131^I and the risk for thyroid cancer has been demonstrated to be dose-dependent [16,17,18,19,20]. For example, a large epidemiological case-control study of Belarusian and Russian children showed a strong dose-dependent increase in the risk for developing thyroid carcinomas, and the risk seemed to increase linearly with the dose in the examined dose range [16]. Recent analysis of the thyroid cancer prevalence in the Belarusian and the Ukrainian Chernobyl cohorts also found a linear dose-response relationship [7,9]. Thus, there is solid evidence that the radiation exposure is a causal factor associated with childhood thyroid cancer.

It is well established that the most prevalent types of thyroid cancers are the papillary and follicular thyroid carcinomas (PTC and FTC, respectively) both in children and adults [20]. After the Chernobyl accident, almost all childhood thyroid cancers were PTCs [8,21]. In earlier cases, a large proportion of the PTCs were of the solid subtype, which was a unique characteristic observed after the Chernobyl accident [8,20]. Subsequently, the growth pattern was shifted to the classic subtype, which is less aggressive and metastatic, and importantly, it is quite common in a sporadic childhood PTC [8,20,21,22]. A recent comparative histological study in the Ukraine cases reported that a dominant papillary growth pattern was less frequent, and an aggressive tumor behavior was more frequent than the sporadic PTCs [23].

## 3. Oncogenic Rearrangements in Childhood Thyroid Cancer

Since sporadic childhood thyroid cancers in the affected areas was quite rare, most cancer cases diagnosed after the Chernobyl accident could be attributable to the radiation exposure. Therefore, thyroid cancers diagnosed in children were expected to provide unique opportunities to scrutinize molecular radiation signatures associated with malignant conversion of the normal thyroid follicular cells [24].

Molecular analyses in early childhood thyroid cancer cases demonstrated a very high prevalence of genome rearrangements between the *Rearranged During Transfection* (*RET)* gene and the *PTC3* gene (*RET/PTC3* rearrangement) located on the same chromosome 10 [25,26,27]. Subsequent studies have shown that the *RET/PTC1* rearrangement is also a common type of oncogenic mutation in the childhood thyroid cancers after the Chernobyl accident [28], and the *RET/PTC* rearrangements are now recognized as predominant driver mutations in both radiation-related and sporadic childhood papillary thyroid cancers [29,30].

The *RET* gene encodes a transmembrane receptor tyrosine kinase, whose endogenous ligand is the glial cell-derived neurotrophic factor (GDNF). The RET protein is principally expressed in the nervous system, and therefore, the expression in the thyroid follicular cells has never been reported. The binding of GDNF, mediated by the GDNF-family receptor-alpha (GFR-alpha), stimulates the receptor dimerization, which is a critical step for activation of the RET tyrosine kinase activity [31]. In thyroid cancers, the 3′ part of the *RET* proto-oncogene, which encodes the kinase domain, is fused to the 5′ regions of various partner genes, which have collectively been designated as the *PTC* genes (Table 1). The *PTC1* gene, also called the *Coiled-Coil Domain Containing 6* (*CCDC6*) gene, as well as the *PTC3* gene, alternatively named the *Nuclear Receptor Coactivator 4* (*NCOA4*) gene, are those ubiquitously expressed in a variety of tissues and organs including thyroid. 

Consequently, the expression of the chimeric *RET/PTC* genes is driven by the promoter of the partner *PTC* genes fused to the *RET* gene, which results in an unscheduled expression of the kinase domain of the RET protein [32,33]. Importantly, the N-terminal parts of the partner proteins commonly possess coiled-coil domains or other structures that enable homodimerization of the RET kinase domain. As a result, the RET/PTC fusion proteins are constitutively active, and stimulate the mitogen-activated protein kinase (MAPK) pathway and other signaling cascades in a ligand-independent way [34,35,36,37]. Other types of rearrangements, such as *ETV6-NTRK3* [38,39] and *STRN-ALK* [40,41], were also identified in the Chernobyl childhood thyroid cancer (Table 1).

The point mutation in the *BRAF* gene, the T1799A, which gives rise to the BRAF^V600E^ protein, is another predominant oncogenic mutation in PTCs, especially in adult cases [20]. The *BRAF* gene mutation and the *RET/PTC* rearrangement show a reciprocal age-association, and in fact, the prevalence of the *BRAF* mutation in childhood thyroid cancers after the Chernobyl accident is below 10% on average [42]. The lowest frequency of the mutant *BRAF* was detected in childhood PTCs developing after the shorter latency, and it likely is increasing in the later-onset tumors in children and young adults, who were exposed to the Chernobyl radiation as children [43].

In relation to the Chernobyl accident, the large-scale ultrasound screening was started after the accident at the Fukushima Daiichi nuclear power plant [44]. The first and second rounds of screening revealed 187 cases diagnosed with the nodules categorized as malignant or suspicious for malignancy among the cohort of approximately 300,000 subjects, covering all children aged 0–18 years old at the time of the accident. Although the cases are very unlikely to be induced by the radiation, driver mutations in thyroid carcinomas were investigated, and the *BRAF^V600E^* mutation was the most prevalent genetic alteration [45].

## 4. Thyroid Cancers among A-Bomb Survivors

Epidemiological studies in the Life Span Study (LSS) cohort of the A-bomb survivors in Hiroshima and Nagasaki, which include approximately 120,000 survivors in Hiroshima and Nagasaki, and the residents who were not in the cities at the time of bombing, have been conducted since 1950 [2]. Periodic reports from the Radiation Effects Research Foundation (RERF) have shown that the radiation exposure to the γ-rays and neutrons increases the risk of cancer mortality and incidence throughout the life. In the early years after the bombing, the risk of leukemia showed a significant increase, and then decreased but not to zero. Thereafter, the incidence and mortality risks for solid cancers started to increase. The excess relative risk for all solid cancer at age 70 years after the exposure at 30 years of age was estimated to be 0.42 per Gy [95% confidence interval: 0.32–0.53] [1]. An increase in the mortality risk was confirmed for cancers of most of the tissues/organs, including the stomach, lung, liver, colon, breast, gallbladder, esophagus, bladder, and ovary, and its dose-response relationship has been reported to be linear. For several cancer types, the risks were higher in the survivors exposed as children [1].

Since thyroid cancer is rarely fatal, the mortality studies have not assessed it. However, the LSS cohort study has demonstrated that the thyroid cancer incidence risk is significantly increased following the radiation exposure [1]. For example, the follow-up of the thyroid cancer incidence in the LSS cohort between 1958 and 2005 estimated the gender-averaged excess relative risk, which is calculated as the relative risk minus one, per Gy as 1.28 [95% confidence interval: 0.59–2.70] at the age of 60 years after the exposure at 10 years of age, although microcarcinoma with a diameter less than 10 mm was not included in the study [46]. The thyroid cancer incidence was strongly dependent on the age-at-exposure, and there was no significant increase in the thyroid cancer incidence among those exposed after the age of 20 [46].

Molecular analyses in the adult-onset PTC cases have demonstrated that more than half of the exposed patients exhibited the *BRAF* point mutation (56%), and the *RET/PTC* rearrangement was observed in 22% of the exposed patients, while more than 80% of the non-exposed cases harbored the *BRAF* gene point mutation [47]. Of importance, there were the opposite trends for the oncogene frequency associated with the radiation dose: An uptrend for *RET/PTC* and downtrend for the *BRAF* mutation. Rearrangements of the *NTRK1* and the *ALK* genes [48], as well as the *ABCD5/RET* rearrangement [49], were also identified.

## 5. Thyroid Cancers among the Patients Who Received External Medical Radiation in Childhood

In addition to the LSS cohort, which includes certain, although limited, number of subjects who were children at the time of the bombings, other groups of externally irradiated children exist. These are patients who received medical radiation of cancer or non-cancer conditions to the head and neck area or to the chest. A pooled analysis of seven independent studies was conducted [10], and more recently, an updated pooled analysis of 12 studies was reported [50], in which the oldest age at exposure was 19, mean five years old. The mean and median doses were 0.71 Gy and 0.07 Gy, respectively, for the range from >0 to 76 Gy.

Across the full dose range, the relative risk for thyroid cancer increased supralinearly for the doses up to 2–4 Gy, leveled at doses between 10 to 30 Gy and declined at higher doses but remained elevated even for the doses exceeding 50 Gy. The relative risk at 1 Gy was 6.5 [95% confidence interval: 5.1–8.5], and increased with a younger age at exposure. The excess relative risk displayed an apparent peak of 20–30 years after exposure and remained elevated after >50 years of the follow-up [50].

The molecular changes in PTCs from the patients, who had received medical external radiation as children, were examined in several studies. In one earlier work, the *RET/PTC* rearrangements were found in 84% (16/19) of radiation-related PTCs, while only 15% (3/20) of prevalence was seen in the sporadic cases [51]. Another study using the Chicago cohort demonstrated that the RET immunoreactivity, which was well correlated with the *RET/PTC* rearrangements, was found in 86.7% of the radiation-exposed cases and in 52.9% of tumors from the control group [52]. Many of these patients received radiotherapy for cancer, so that the doses were relatively high compared with the tinea capitis patients. Moreover, the distribution of age at diagnosis was much younger in the irradiated cases, which might have influenced the mutation spectrum [53].

## 6. Radiation Signatures and Possible Mechanism of Radiation Carcinogenesis

**Figure 1:**
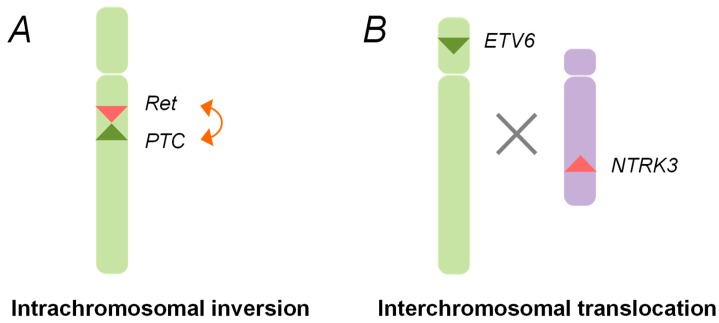
Schematic representation of oncogenic chromosomal rearrangements. (**A**) Intra-chromosomal inversion. The *rearranged during transfection* (*RET)* gene and the *PTC1/3* gene (*RET/PTC*) rearrangements are generated by an intra-chromosomal inversion of chromosome 10, which gives rise to the fusion genes between the tyrosine kinase domain of the *RET* gene and the amino terminal region of the *PTC* gene; (**B**) Inter-chromosomal translocation. The chromosomal rearrangements, such as ETV6-NTRK3, are created through an illegitimate recombination between the different broken chromosomes.

After the Chernobyl accident, chromosomal rearrangements, such as the *RET/PTC1* and *RET/PTC3*, were identified in childhood thyroid cancers. These rearrangements are generated through the paracentric (intrachromosomal) inversion within the long arm of chromosome 10, where the *RET*, the *CCDC6,* and the *NCOA4* genes are located (Figure 1) [29,30,36]. The inter-chromosomal translocation is also involved in the formation of other types of rearrangements, such as *ETV6-NTRK3* (Figure 1).

Theoretically, rearrangements need at least two DNA double-strand breaks, so that exposure to the radiation, which is a well-known inducer for DNA double-strand breaks, has been assumed to cause such rearrangements through an illegitimate recombination of the broken DNA ends [24]. Furthermore, it has been proposed that the folding of the chromosomal 10q11.2–21 region facilitates a spatial proximity of the *RET* and *PTC* genes, which could be a structural basis for the RET/PTC rearrangements [54,55]. The close connection between the radiation exposure and the induction of chromosomal rearrangement was demonstrated experimentally. For example, the *RET/PTC* rearrangements were detected in the X-irradiated primary thyroid cells and tissues [56,57]. While the initial studies used a high-dose over 50 Gy, the generation of the *RET/PTC* rearrangements were also identified in the thyroid epithelial cells receiving lower doses [58]. The induction of other chromosomal rearrangements has also been demonstrated in vitro [39]. 

Although the experiments have proven that the *RET/PTC* rearrangements are induced by the radiation exposure, in vitro studies are unable to evaluate a spontaneous incidence of the *RET/PTC* rearrangements, as the frequency of those in the absence of the genotoxic stimuli is too low. Therefore, the information from human studies, which analyzed the *RET/PTC* rearrangements in sporadic childhood thyroid cancers, is indispensable. While several independent groups have evaluated the prevalence of the *RET/PTC* rearrangements in childhood thyroid cancer after the Chernobyl accident, only some studies have compared the results with the frequency of the *RET/PTC* rearrangements in sporadic childhood PTCs [27,38,43,59]. The compiled data demonstrated that except for the *RET/PTC3* in tumors developing within the first decade after the Chernobyl accident, the frequency of rearrangements, in particular that of the *RET/PTC1* rearrangement, was comparable between childhood thyroid cancers after the Chernobyl accident and those occurring irrespectively of the radiation exposure (Table 2) [42,60,61,62,63]. This suggests that the *RET/PTC* rearrangements in radiation-related cases might not be the radiation signature. Rather, the radiation exposure could unveil the *RET/PTC* rearrangements that occurred spontaneously [24]. Considering that thyroid cancers in children began to manifest 4–5 years after the Chernobyl accident, it would be reasonable to hypothesize that thyroid follicular cells with the *RET/PTC* rearrangements already existed, and the radiation exposure could provide a chance for the cells with such cancer signatures to proliferate [24].

It is well-documented that *RET/PTC1* is the predominant type of gene rearrangements in the pediatric PTC [64,65,66,67], and that the frequency of sporadic thyroid cancer cases harboring the *RET/PTC* rearrangements decreases with age, while those with the *BRAF* mutation becomes more common [20]. These two genetic changes are mutually exclusive. Individuals born before the accident are now at least thirty-three years old, and recent reports demonstrate that the frequency of thyroid cancer driven by the *BRAF* mutation tends to grow in the affected group [68,69]. This is an important observation indicating that molecular changes in the radiation-related thyroid cancer mirror those occurring spontaneously, although we also need to bear in mind that there were studies reporting a decrease of the *RET/PTC* rearrangements over the years in adult PTCs [70,71].

Thus, the spectrum of genetic alterations identified in thyroid cancers related to the Chernobyl accident are not very different from that found in the sporadic cases. Since exposures to natural reactive oxygen species and environmental chemicals may occur any time during the life, including the in utero period, it cannot even be ruled out that Chernobyl childhood PTCs could originate from the thyroid follicular cells that had already carried spontaneous *RET/PTC* rearrangements before the exposure.

Undoubtedly, there is considerable evidence of a link between the chromosomal rearrangements and radiation dose. For example, a recent publication analyzed driver mutations in a series of 65 PTCs diagnosed after the Chernobyl accident, with the individual doses available [41]. Chromosomal rearrangements, including *RET/PTC1*, *ETV6-NTRK3*, *STRN-ALK*, and *RET/PTC3*, and point mutations, such as BRAF^V600E^, were found in 70.8% and 26.2% of the cases, respectively. A significant positive correlation between the ^131^I thyroid dose and the incidence of chromosomal rearrangements was found, and the study reasonably claimed these could be induced by the radiation exposure. The dose-dependent incidence of the gene rearrangement was also reported for the *ETV6-NTRK3* and *STRN-ALK* rearrangements [39,40]. By contrast, other reports concluded that the prevalence of the *RET/PTC* rearrangements were not associated with the exposure [72] or individual radiation doses [73]. The dose-dependency of *RET/PTC* and *PAX8/PPARγ*, which is the fusion occurring in the follicular thyroid carcinoma, between PAX8, a transcription factor involved in the thyroid development and PPAR*γ*, the master transcriptional regulator of adipogenesis [74,75], was also determined in radiation-related Chernobyl cases but the decline in the rearrangement frequency at higher doses was modelled and the confidence interval was very wide [76].

Recent studies have analyzed various molecular changes besides the chromosomal rearrangements [44]. For example, certain differences in the gene expression profiles between radiation-related and sporadic cancers were reported, although there is a lack of consistency between the gene signatures. Possible confounding factors, including pathological features of the tumors, could cause discrepancies between the studies, and the gene signatures might reflect the results of the radiation exposure. In addition, there has been a gain of chromosome 7q11.23, where the *CLIP2* gene is located, associated with radiation-related cases [77,78]. ClIP2 (CAP-Gly domain containing linker protein 2) is a member of the cytoplasmic linker protein families, which might link organelles with microtubules. The CLIP2 protein also contains a SMC (structural maintenance of chromosomes) domain involved in the chromosome segregation and cell division. Its overexpression could be a marker of the radiation etiology of thyroid cancer, however, an involvement into the molecular mechanisms of radiation-induced thyroid carcinogenesis needs to be established. Furthermore, genetic determinants connected to the individual predisposition to thyroid cancer were reported. Genome-wide association studies using Chernobyl cases have identified common single nucleotide polymorphism markers, such as rs965513, located in the *FOXE1* region, while there is no marker specific for the radiation-related cancers [79]. Thus, analyses with advanced technologies are necessary to obtain more information on molecular structures, which should be needed for determining molecular radiation signatures in childhood thyroid cancers related to the radiation exposure.

## 7. Conclusions

Internal exposure to the radioactive iodine as well as the external radiation exposure increase the risk for childhood papillary thyroid cancer. Molecular analyses have shown that *RET/PTC* and other gene rearrangements are the most prevalent oncogenic alteration in both the radiation-induced and sporadic childhood thyroid cancer. While the contribution of the radiation exposure to the induction of oncogenic rearrangements in PTC in exposed patients still needs to be clarified, further molecular approaches are expected to provide clues to untangle our debates on the role of radiation exposure in the development of childhood thyroid cancer.

## Figures and Tables

**Table 1:** Oncogenic rearrangements in childhood thyroid cancers related to the Chernobyl accident.

Oncogenes	Rearrangement Partners	Chromosome Location	Type of Rearrangements
RET rearrangements			
*RET/PTC1*	*CCDC6* (also *H4*)	10q11.21/10q21	Inversion
*RET/PTC2*	*PRKAR1A*	10q11.21/17q24.2	Translocation
*RET/PTC3*	*NCOA4* (also *Ele*)	10q11.21/10q11.22	Inversion
*RET/PTC4*	*NCOA4* (also *Ele*)	10q11.21/10q11.22	Inversion
*RET/PTC5*	*GOLGA5* (also *RFG5*)	10q11.21/14q32.12	Translocation
*RET/PTC6*	*TRIM24*	10q11.21/7q32-q34	Translocation
*RET/PTC7*	*TRIM33* (also *RFG7*)	10q11.21/1p13.1	Translocation
*RET/PTC8*	*KTN1*	10q11.21/14q22.1	Translocation
*RET/PTC9*	*RFG9* (also *MBD1*)	10q11.21/18q21	Translocation
SPECC1L-RET	SPECC1L	22q11.23/10q11.21	Translocation
SQSTM1-RET	SQSTM1	5q35.3/10q11.21	Translocation
BRAF rearrangements			
*AKAP9/BRAF*	*AKAP9*	7q21.2/7q34	Inversion
A*GK/BRAF*	*AGK*	7q34/7q34	Inversion
SND1-BRAF	SND1	7q32.1/7q34	Inversion
MBP-BRAF	MBP	18q23/7q34	Translocation
POR-BRAF	POR	7q11.23/7q34	Inversion
ZBTB8A-BRAF	ZBTB8A	1p35.1/7q34	Translocation
MACF-BRAF	MACF1	1p34.3/7q34	Translocation
NTRK rearrangements			
*TPR/NTRK1*	*TPR*	1q31.1/1q23.1	Inversion
BANP-NTRK1	BANP	16q24.2/1q23.1	Translocation
*ETV6/NTRK3*	*ETV6*	12p13.1/15q25.3	Translocation
PPARg rearrangements			
*PAX8/PPARg*	*PAX8*	2q14.1/3p25.2	Translocation
*CREB3L2/PPARg*	*CREB3L2*	7q33/3p25.2	Translocation
Other rearrangements			
STRN-ALK	*ALK*	2p22.2/2p23.2-p23.1	Inversion
THADA-IGF2BP3		2p21/7p15.3	Translocation

**Table 2:** Prevalence of oncogenic mutations in childhood papillary thyroid carcinomas.

Studies	Prevalence (Positive Cases/Total (%))		
	RET/PTC1 Rearrangement		Ref
	Chernobyl-related	Sporadic	
Nikiforov et al. (1997)	5/22	6/14	27
Thomas et al. (1999)	12/63		61
Rabes et al. (2000)	40/172		62
Elisei et al. (2001)	6/25	5/25	63
Ricarte-Filho et al. (2013)	3/18	1/18	38
Leeman-Neill et al. (2013)	14/62		76
Total	80/362 (22.1)	12/57 (21.1)	
